# Supplementary material for: Evaluating Known Zika Virus NS2B-NS3 Protease Inhibitor Scaffolds via In Silico Screening and Biochemical Assays
Source: Pharmaceuticals (Basel). 2023 Sep 19;16(9):1319. doi: 10.3390/ph16091319 (PMC10537087; doi:10.3390/ph16091319)
Supplement: Supplementary file 1 [file pharmaceuticals-16-01319-s001.zip › pharmaceuticals-2570112-supplementary.pdf]

Supplementary Material

Evaluating known Zika virus NS2B-NS3 protease inhibitor scaffolds by in silico screening and biochemical assays

Lucianna H. Santos [1,‡], Rafael E. O. Rocha [1], Diego L. Dias [2], Beatriz M. R. M. Ribeiro [1], Mateus Sá M. Serafim [2], Jônatas S. Abrahão [2] and Rafaela S. Ferreira [1,*]

[1] Departamento de Bioquímica e Imunologia, Instituto de Ciências Biológicas, Universidade Federal de Minas Gerais (UFMG), Belo Horizonte, 31270-901, MG, Brazil.

[2] Departamento de Microbiologia, Instituto de Ciências Biológicas, Universidade Federal de Minas Gerais (UFMG), Belo Horizonte, 31270-901, MG, Brazil.

‡ Current address: Institut Pasteur de Montevideo, Mataojo 2020, 11400, Montevideo, Uruguay.

* Correspondence: rafaelasf@icb.ufmg.br

Table S1. Number of compounds found on ZINC15 that have any similarity with Lee et al. (2017) inhibitors.

Query	ZINC IDs	N° of compounds*
Scaffold	**	102
LEE-1	ZINC000009325712	41
LEE-2	ZINC000020570103	158
LEE-3	ZINC000009325709	68
LEE-4	ZINC000009354775	158
LEE-5	ZINC000009354778	44
LEE-6	ZINC000009404221	8
LEE-7	ZINC000009404670	9
LEE-8	ZINC000009325713	27
LEE-9	None found	3
LEE-10	None found	0
Total	-	615
Without duplicates***	-	509

* The compounds were queried using the 2019 version of ZINC 15, employing the Substructure and Tanimoto similarity options. ** The scaffold identified for LEE-1, LEE-2, LEE-3, LEE-4, and LEE-8 was FTFINDUAXQKBPQ-UHFFFAOYSA-N. For LEE-5, the scaffold identified was QRBWCVPETSVZCW-UHFFFAOYSA-N. LEE-6 had the scaffold ZCVZIZAPLBGIBR-UHFFFAOYSA-N, while LEE-7 had the scaffold HSGVUNDOGWMDOE-UHFFFAOYSA-N. *** Individual searches identified duplicates. We removed these duplications.

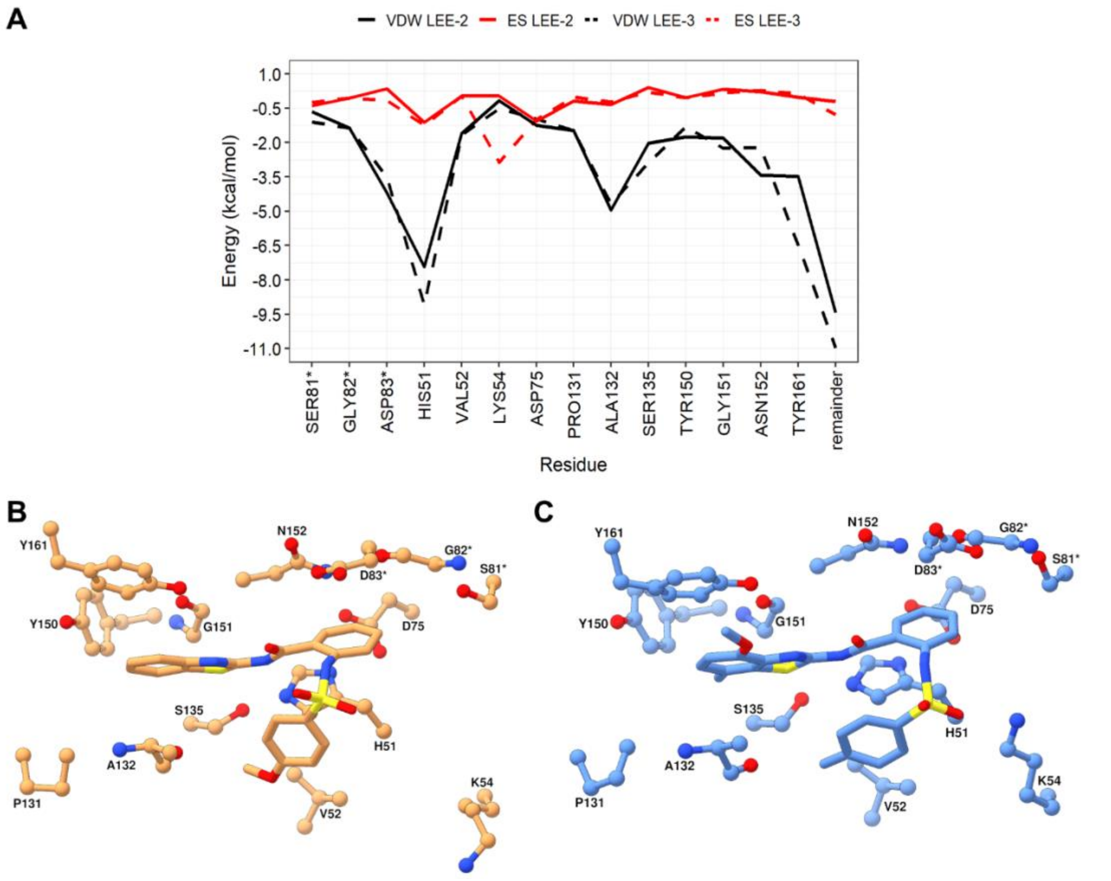

Figure S1. Interaction signature and putative binding modes of LEE-2 and LEE-3. (A) Per-residue van der Waals (VDW, black lines) and electrostatic (ES, red lines) interaction energies (kcal/mol) of the reference compounds LEE-2 (solid lines) and LEE-3 (dashed lines). Interaction energies are almost superimposed due to the similar binding modes. Binding modes of LEE-2 (B) and LEE-3 (C) from Santos et al. (2022).

Table S2. Zinc ID, ID, score, and hydrogen bond (hbond) count for the selected 36 compounds.

ZINC ID	ID	MULTIGRID SCORE	HBOND (ALL)	HBOND LIGAND ATOMS	HBOND PROTEASE ATOMS
ZINC001638119710		-47.70	1	1	1
ZINC001627271434		-46.49	1	1	1
ZINC001611309804	5	-46.34	1	1	1
ZINC001294554668		-45.46	1	1	1
ZINC000254402523		-44.67	1	1	1
ZINC001540153846		-42.98	1	1	1
ZINC001471637015	12	-42.77	1	1	1
ZINC000254523810	9	-42.68	1	1	1
ZINC000254820807		-42.59	2	2	2
ZINC000760952832	4	-42.28	1	1	1
ZINC000006147289		-42.08	2	2	2
ZINC000426623750	1	-42.01	1	1	1
ZINC000254744176		-41.58	1	1	1
ZINC001469953631		-41.57	1	1	1
ZINC000254555735	11	-41.47	1	1	1
ZINC001650929045		-41.07	1	1	1
ZINC000510134491	7	-40.80	1	1	1
ZINC001301832358		-40.77	1	1	1
ZINC000760949985	3	-40.73	1	1	1
ZINC000506553351		-40.63	1	1	1
ZINC001631305868		-40.20	1	1	1
ZINC000100873004	10	-40.15	2	2	2
ZINC000487872991		-39.89	1	1	1
ZINC001545875234		-39.85	1	1	1
ZINC001639199761		-39.77	1	1	1
ZINC000479639818	2	-39.35	1	1	1
ZINC000511552346		-39.22	1	1	1
ZINC000009471842		-39.15	1	1	1
ZINC000006146757		-39.13	1	1	1
ZINC001635657618		-38.63	1	1	1
ZINC000020569683		-38.40	1	1	1
ZINC000519475921	8	-38.33	1	1	1
ZINC001135702587	6	-37.42	1	1	1
ZINC001637550049	13	-36.71	1	1	1
ZINC000427717744		-35.93	1	1	1
ZINC000507148232		-34.95	1	1	1

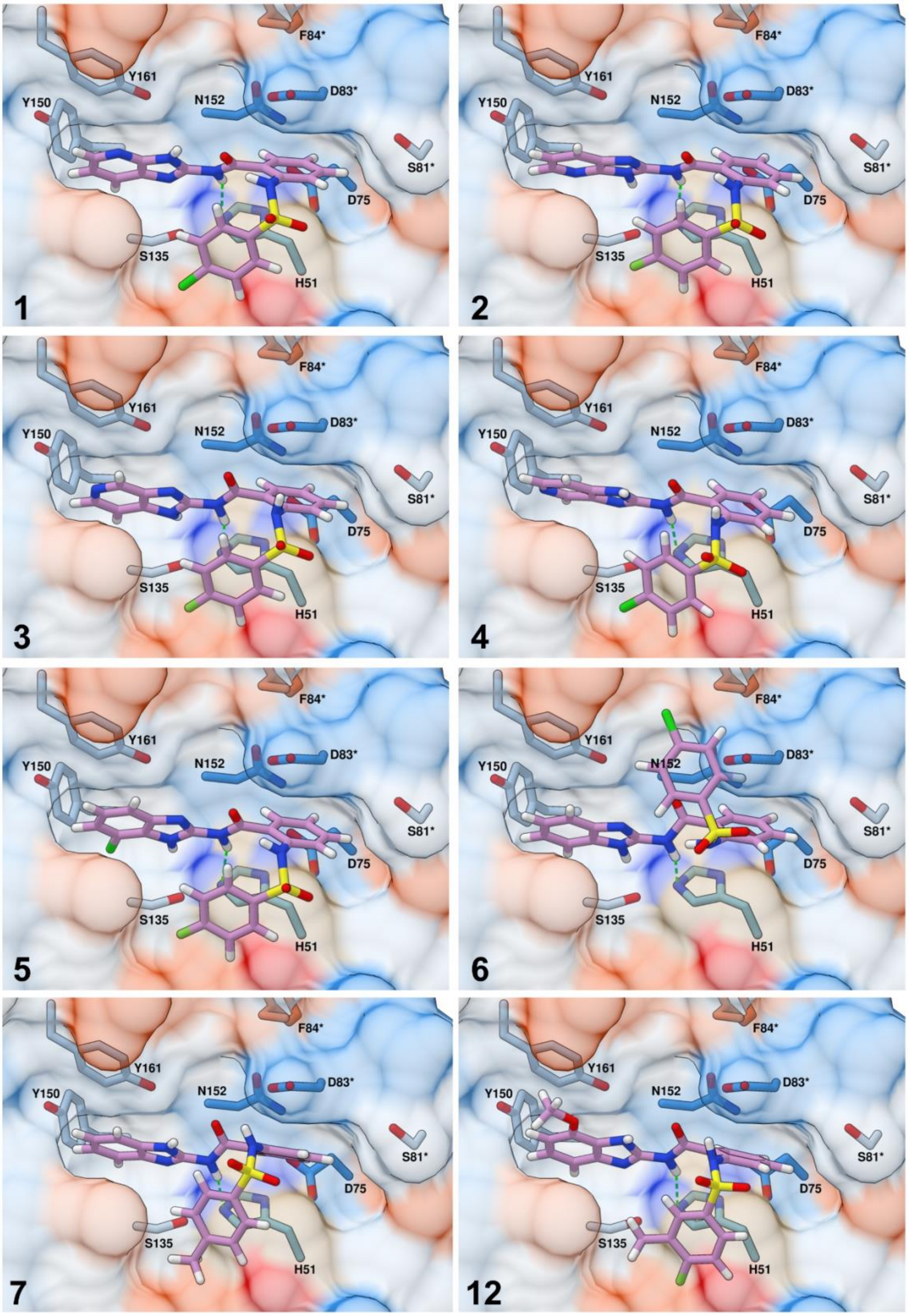

Figure S2. Binding modes of chosen compounds **1**, **2**, **3**, **4**, **5**, **6**, **7**, and **12** from molecular docking. Surface and NS2B-NS3pro residues are colored by hydrophobicity. Colored areas range from dodger blue for the most hydrophilic, to orange red for the most hydrophobic.

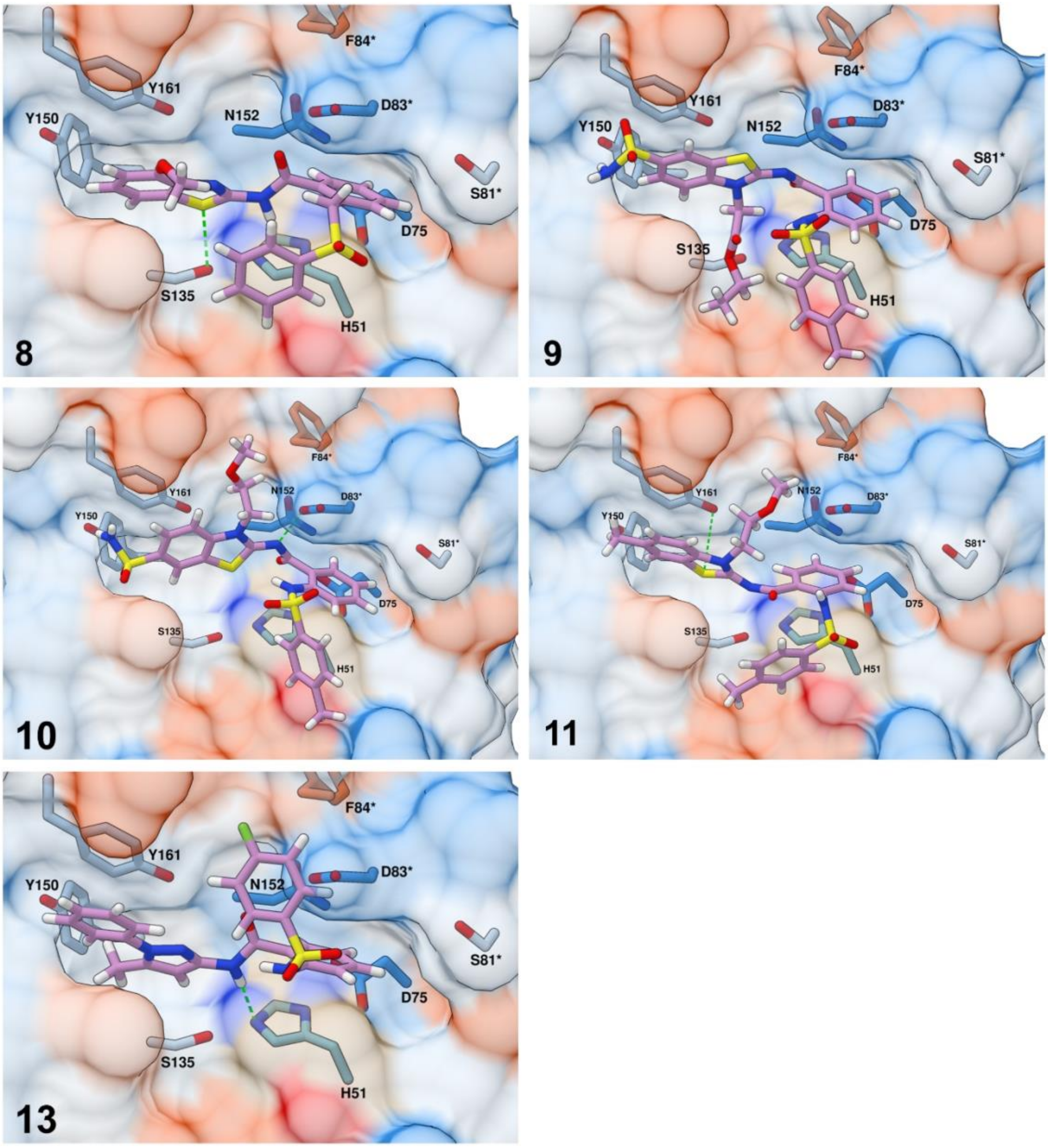

Figure S3. Binding modes of chosen compounds **8**, **9**, **10**, **11**, and **13** from molecular docking. Surface and NS2B-NS3pro residues are colored by hydrophobicity. Colored areas range from dodger blue for the most hydrophilic, to orange red for the most hydrophobic.

Table S3. Hill coefficient retrieved from dose-response curves for the most potent ZIKV inhibitors.

Hill Slope		
ID	Replica 1	Replica 2
1	3.046	1.585
2	0.731	4.089
4	0.8047	0.5347
5	3.333	1.731
6	6.697	4.362
7	2.014	1.056
12	1.517	1.742
13	5.057	5.334

References

Lee, H., Ren, J., Nocadello, S., Rice, A. J., Ojeda, I., Light, S., Minasov, G., Vargas, J., Nagarathnam, D., Anderson, W. F., & Johnson, M. E. (2017). Identification of novel small molecule inhibitors against NS2B/NS3 serine protease from Zika virus. *Antiviral Research*, *139*, 49–58. https://doi.org/10.1016/j.antiviral.2016.12.016

Santos, L. H., Caffarena, E. R., & Ferreira, R. S. (2022). pH and non-covalent ligand binding modulate Zika virus NS2B/NS3 protease binding site residues: Discoveries from MD and constant pH MD simulations. *Journal of Biomolecular Structure and Dynamics*, *40*(20), 10359–10372. https://doi.org/10.1080/07391102.2021.1943528
